# Repurposing weather modification for cloud research showcased by ice crystal growth

**DOI:** 10.1093/pnasnexus/pgae402

**Published:** 2024-09-18

**Authors:** Fabiola Ramelli, Jan Henneberger, Christopher Fuchs, Anna J Miller, Nadja Omanovic, Robert Spirig, Huiying Zhang, Robert O David, Kevin Ohneiser, Patric Seifert, Ulrike Lohmann

**Affiliations:** Department of Environmental Systems Science, Institute for Atmospheric and Climate Science, ETH Zurich, Zurich 8092, Switzerland; Department of Environmental Systems Science, Institute for Atmospheric and Climate Science, ETH Zurich, Zurich 8092, Switzerland; Department of Environmental Systems Science, Institute for Atmospheric and Climate Science, ETH Zurich, Zurich 8092, Switzerland; Department of Environmental Systems Science, Institute for Atmospheric and Climate Science, ETH Zurich, Zurich 8092, Switzerland; Department of Environmental Systems Science, Institute for Atmospheric and Climate Science, ETH Zurich, Zurich 8092, Switzerland; Department of Environmental Systems Science, Institute for Atmospheric and Climate Science, ETH Zurich, Zurich 8092, Switzerland; Department of Environmental Systems Science, Institute for Atmospheric and Climate Science, ETH Zurich, Zurich 8092, Switzerland; Department of Geosciences, Section for Meteorology and Oceanography, University of Oslo, Oslo 0316, Norway; Department Remote Sensing of Atmospheric Processes, Leibniz Institute for Tropospheric Research (TROPOS), Leipzig 04318, Germany; Department Remote Sensing of Atmospheric Processes, Leibniz Institute for Tropospheric Research (TROPOS), Leipzig 04318, Germany; Department of Environmental Systems Science, Institute for Atmospheric and Climate Science, ETH Zurich, Zurich 8092, Switzerland

**Keywords:** clouds, ice crystals, cloud seeding, airborne observations, radar observations

## Abstract

The representation of cloud processes in models is one of the largest sources of uncertainty in weather forecast and climate projections. While laboratory settings offer controlled conditions for studying cloud processes, they cannot reproduce the full range of conditions and interactions present in natural cloud systems. To bridge this gap, here we leverage weather modification, specifically glaciogenic cloud seeding, to investigate ice growth rates within natural clouds. Seeding experiments were conducted in supercooled stratus clouds (at −8 to $-5^{\circ }$C) using an uncrewed aerial vehicle, and the created ice crystals were measured 4–10 min downwind by in situ and ground-based remote sensing instrumentation. We observed substantial variability in ice crystal growth rates within natural clouds, attributed to variations in ice crystal number concentrations and in the supersaturation, which is difficult to reproduce in the laboratory and which implies faster precipitation initiation than previously thought. We found that for the experiments conducted at $-5.2^{\circ }$C, the ice crystal populations grew nearly linearly during the time interval from 6 to 10 min. Our results demonstrate that the targeted use of weather modification techniques can be employed for fundamental cloud research (e.g. ice growth processes, aerosol–cloud interactions), helping to advance cloud microphysics parameterizations and to improve weather forecasts and climate projections.

**Significance Statement**

The rate at which ice crystals grow in the atmosphere plays a crucial role in precipitation formation and the Earth’s climate system. However, it has been challenging to systematically study ice crystals in natural clouds. Here, we use weather modification to infer the growth rates of ice crystals in a natural cloud environment. We find that the observed ice crystal growth rates have a considerable larger variability than in previously published laboratory studies, which can potentially accelerate precipitation initiation. The methodology proposed herein will advance our understanding in cloud physics and has the potential to revolutionize the parameterization of cloud processes in weather and climate models.

## Introduction

The concentration and size of ice crystals in clouds have a significant influence on precipitation formation (1, 2), the radiative energy budget (3), and cloud lifetime (4). Despite their important role, fundamental knowledge gaps in ice formation and growth processes exist, owing to complex interactions among the hydrometeors and the wide variety of ice crystal shapes and densities found in nature (5, 6). Understanding and quantifying the growth of ice crystals in the atmosphere is crucial for developing physically based parameterizations in weather and climate models (7).

At temperatures above $-38^{\circ }$C, a special subset of aerosols known as ice nucleating particles are required for ice crystals to form (8). Once formed, ice crystals grow by vapor deposition into their preferred ice habit at the given ambient temperature and relative humidity. The vapor attaches to the crystal surface in steps centered on the corners, where the supersaturation is highest, and then spreads as layers along the faces (9–11). While temperature determines the main growth direction by controlling the relative rates of surface diffusion on the basal and prism faces (10, 12, 13), supersaturation determines the magnitude of the growth rate and the degree of secondary growth features such as hollows (9, 14–17). Furthermore, ice crystals can grow at the expense of evaporating cloud droplets when the vapor pressure lies between ice ($e_{i}$) and water saturation ($e_{s})$ (i.e. $e_{i}<e_{s}$) (18, 19), a process referred to as the Wegener–Bergeron–Findeisen (WBF) process (20–22). The speed of the WBF process strongly depends on whether cloud droplets and ice crystals are uniformly distributed (genuinely mixed) or spatially separated (conditionally mixed) within a cloud volume (23). As ice crystals grow, they can collide and coalesce with other hydrometeors (e.g. riming, aggregation) or splinter and produce new ice crystals through secondary ice production processes (24). However, like microphysics in general, ice processes cannot be resolved in weather and climate models and need to be parameterized. Improper representation of ice processes in models introduces uncertainties in both weather forecasts and climate projections (7, 19, 25, 26).

Microphysical parameterizations are developed based on both theory and observations from laboratory and field studies (27). These parameterizations consist of simplified rate equations that are tailored to represent a specific microphysical process. However, these process rate parameterizations, such as the vapor diffusional growth rate of ice crystals, are difficult to constrain. The only way to quantify individual process rates under controlled conditions is through laboratory experiments. For example, Ryan et al. (28) measured the axial and mass growth rates of freely suspended ice crystals in a cloud chamber at temperatures ranging from $-21^{\circ }$C to $-3^{\circ }$C for growth times of up to 3 min. Similarly, Takahashi et al. (29) measured the vapor diffusional growth rate of ice crystals for extended growth periods of up to 30 min in a vertical supercooled cloud tunnel at temperatures ranging between $-23^{\circ }$C and $-3^{\circ }$C. Both studies observed the maximum growth rates on the basal face at around $-6^{\circ }$C (i.e. along *c*-axis, columnar growth) and on the prism face at $-15^{\circ }$C (i.e. along *a*-axis, plate-like growth). While laboratory studies are crucial for studying processes within a well-controlled environment, they have difficulty exploring the full range of atmospheric conditions present in natural clouds due to design limitations (e.g. short residence time) and the constrained setting (e.g. limited variability in temperature and/or supersaturation).

Up to now, it has been challenging to use field observations to constrain microphysical parameterizations, given the simultaneous occurrence of multiple interacting processes in natural clouds and the significant temporal and spatial variability. This necessitates the use of sophisticated sampling strategies that go beyond measuring the current state of the cloud. Possible approaches include cloud measurements along quasi-Lagrangian flight tracks (30–32) or repeated penetrations in quasi-steady-state clouds such as wave clouds (33, 34). These studies were limited to temperatures below $-10^{\circ }$C, where clouds contain sufficiently high concentrations of naturally occurring ice nucleating particles (8), to obtain solid statistics. To address this lack of observations, here we use glaciogenic cloud seeding (i.e. injection of artificial ice nucleating particles) to produce ice crystals and initiate subsequent growth processes in supercooled stratus clouds otherwise devoid of ice (see *Methods*). By adjusting the distance and time between seeding and measurement, we can quantify the ice crystal growth rates across a range of growth times and temperatures. This approach allows growth rate studies to extend to temperatures above $-10^{\circ }$C, a temperature range that is particularly important, as it covers crucial precipitation initiation processes (e.g. WBF, seeder–feeder, Hallett-Mossop).

## Results

**Fig. 1.**
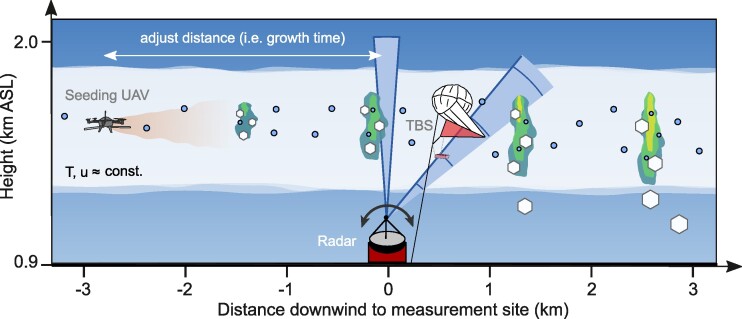
Schematic of the experimental setup of CLOUDLAB. Seeding particles (brown plume) are released into a supercooled stratus cloud by a UAV flying tracks perpendicular to the wind. This triggers ice formation and growth via the WBF process, as illustrated by the increase in the ice (white hexagons) and the depletion of the liquid phase (blue circles). The seeding signal is measured downwind by a tethered balloon system and by vertically pointing and scanning cloud radars (blue). The expected radar seeding reflectivities are highlighted by blue–green–yellow colors. The seeding distance (and thus ice growth time) is varied between consecutive seeding missions, while the environmental parameters such as temperature (*T*) and wind speed (*u*, from left to right) remain approximately constant, enabling systematic studies on ice growth.

The observations presented here were obtained during the CLOUDLAB measurement campaigns (35) conducted in the Swiss Plateau, where wintertime stratus clouds were seeded by a burn-in-place flare mounted on an uncrewed aerial vehicle (UAV) to trigger ice formation and subsequent ice growth processes. The seeding-induced microphysical changes were observed 4 to 10 min downwind of the seeding location by ground-based remote sensing instrumentation and in situ instrumentation (e.g. holographic imager HOLIMO) mounted on a tethered balloon system (36). One seeding experiment included several consecutive seeding missions (conducted within 30 min of each other) with changing distance (2–3 km) between seeding and measurement location. The environmental conditions were assumed to remain approximately constant during this time period due to the persistent nature of stratus clouds (37). Following this approach, systematic ice growth studies were conducted in this natural quasi-steady-state environment by adjusting the seeding distance (and consequently ice growth times) and measuring the resulting ice crystal sizes. A conceptual overview of the observational setup and experimental approach is shown in Fig. 1 and described in more detail in the Methods section.

**Fig. 2.**
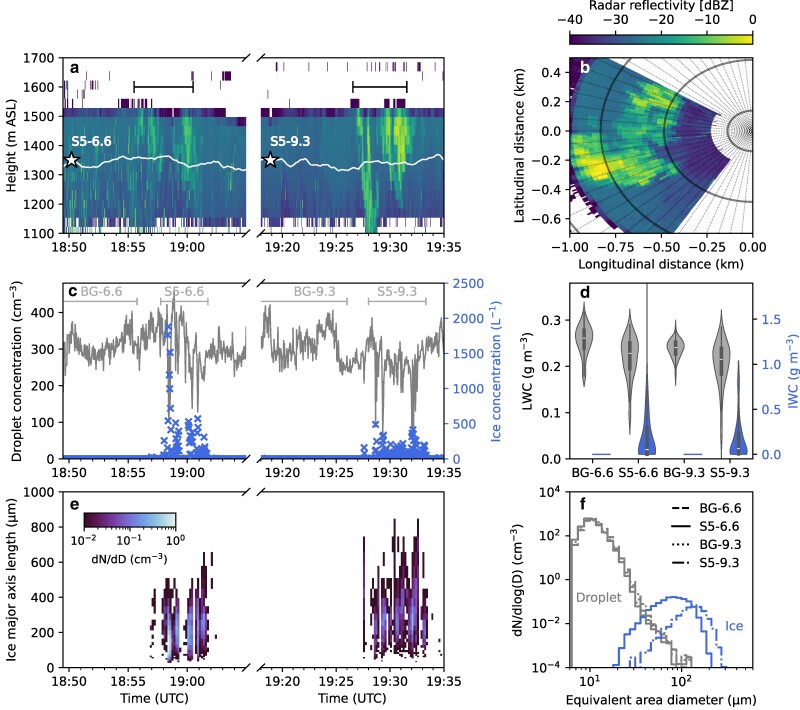
Temporal evolution of the microphysical properties observed during the seeding missions S5-6.6 and S5-9.3 conducted on 2023 January 24 at a seeding temperature of $-5.2^{\circ }$C and at seeding distances of 2 and 3 km, respectively. a) Time–height radar reflectivity measured by the vertically pointing W-band cloud radar. The white line shows the height of the tethered balloon, whereas the white stars indicate the height and start time of the seeding missions. The black bars highlight the time periods of expected seeding signal, taking into account the mean wind speed and the flare burning time of 5 min. b) Radar reflectivity measured by the scanning Ka-band cloud radar during a plan position indicator (PPI) scan at 19:31 UTC at an elevation angle of 30° and an azimuth angle ranging between 212° and 302°. c) Timeseries of the cloud droplet number concentration (gray line) and ice crystal number concentration (blue crosses). The background periods (BG-6.6: 18:45:45–18:55:45 UTC, BG-9.3: 19:16:00–19:26:00 UTC) and seeding periods (S5-6.6: 18:57:45–19:01:45 UTC, S5-9.3: 19:28:00–19:33:20 UTC) are highlighted by the horizontal gray bars. d) Violin plots of the liquid water content (LWC, gray) and ice water content (IWC, blue) measured during the background and seeding periods (same periods as depicted in c). Note that no ice crystals were measured during the background periods (i.e. IWC=0). e) Timeseries of the ice crystal size distributions in terms of the major axis length. f) Cloud droplet size distribution (gray) and ice crystal size distribution (blue) measured during BG-6.6 (dashed), S5-6.6 (solid), BG-9.3 (dotted), and S5-9.3 (dot-dashed) in terms of the equivalent area diameter. The data presented in c)–f) were obtained from HOLIMO and averaged over 2 s c,d) and 10 s e), respectively.

Two seeding experiments conducted at different seeding temperatures are shown in Fig. 2 ($\text{T}=-5.2^{\circ }$C) and Fig. S1 ($\text{T}=-7.2^{\circ }$C). The experimental parameters of the seeding missions are summarized in Table 1. For both sets of experiments, the seeding material was introduced 2 km and 3 km upwind of the observational site by the UAV, leading to different growth times. The seeding pattern consisted of four 400 m horizontal legs at constant altitude perpendicular to the wind direction within the flare burning time of 5 min.

**Table 1.** Experimental parameters of the seeding missions.

Name	ID	Date (UTC)	*T* (°C)	*d* (km)	*t* (min)
S5-6.6	SM055	24 Jan 2023 18:50	− 5.2	2.0	6.6
S5-9.3	SM056	24 Jan 2023 19:19	− 5.2	3.0	9.3
S7-4.9	SM075	27 Jan 2023 16:25	− 7.2	2.0	4.9
S7-6.2	SM074	27 Jan 2023 16:00	− 7.2	3.0	6.2

Listed are the mission name (consisting of temperature and growth time), CLOUDLAB mission ID, the date and time of the seeding mission, the seeding temperature (*T*), the distance (*d*) between the seeding and measurement location, and the ice crystal growth time (*t*) assuming immediate nucleation after seeding (see Methods).

Here, we provide a comprehensive description of the seeding-induced microphysical changes for the experiment conducted at a temperature of $-5.2^{\circ }$C (Fig. 2), but the findings also remain applicable for the experiment conducted at $-7.2^{\circ }$C (Fig. S1). The natural background cloud on 2023 January 24 was characterized by a radar reflectivity of −25 dBZ, a cloud droplet concentration of 250–350 ${\text{cm}}^{-3}$, a liquid water content of 0.2–0.25 g m^−3^, and a low ice water content of <0.001 g m^−3^ (Fig. 2a,c,d). During the passage of the seeding plume, the radar observations indicated regions of locally enhanced radar reflectivities of up to 0 dBZ (Fig. 2a,b), which clearly stand out from the natural background. Although the seeding material was injected at a constant altitude, seeding signatures were observed across a large vertical extent in the cloud radar due to turbulent mixing, updrafts induced from latent heat release, and sedimentation of ice crystals (38). HOLIMO revealed high concentrations of ice crystals (up to 2,000 L^−1^) with a simultaneous reduction in cloud droplet number concentration during the passage of the seeding plume (Fig. 2c). During some time periods, the liquid phase was entirely depleted, demonstrating the efficiency of the WBF process in natural clouds. After the passage of the seeding plume, the cloud properties returned to the natural background levels present prior to seeding. Thus, the crucial assumption of constant microphysical and environmental properties in the background between consecutive seeding missions was substantiated by observations. The ice crystals measured during S5-9.3 were consistently larger than the ice crystals measured during S5-6.6, due to the longer growth time (Fig. 2e,f). This increase in ice crystal size was also visible in the enhanced radar reflectivities during S5-9.3 (Fig. 2a). Thus, by employing glaciogenic cloud seeding in supercooled stratus clouds, we were able (i) to produce a sufficient number of ice crystals at temperatures above $-10^{\circ }$C, ensuring robust statistics and (ii) to systematically adjust the growth time (by changing seeding distance) between consecutive seeding missions, resulting in different ice crystal sizes, allowing for comprehensive growth studies in natural clouds.

**Fig. 3.**
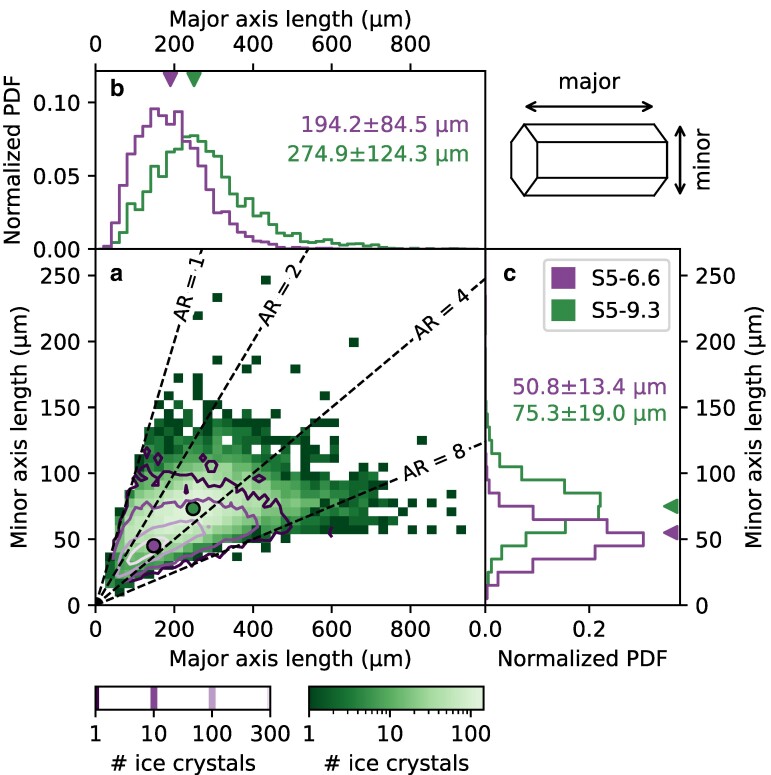
Overview of the ice crystal axial dimensions measured during the seeding missions S5-6.6 (N=17,796) and S5-9.3 (N=7,041). a) 2D histogram of the major and minor axis length of the detected ice crystals. The number of ice crystals observed with a given major and minor axis length during S5-9.3 are shown with the green colormap, while S5-6.6 is shown by the purple contour lines. The dots represent the corresponding median axial dimensions (including all observed ice crystals) and the black lines show contour lines of aspect ratio (defined by major axis length divided by minor axis length). b, c) Normalized probability density function (PDF) of the major and minor axis length. The triangles represent the corresponding time-weighted medians, whereas the numbers indicate the mean values and standard deviations of the PDFs. In the upper-right corner, a schematic of a column’s major and minor axes is displayed, corresponding to growth along the basal and prism faces.

Given that the shape of ice crystals resulting from growth by vapor deposition is influenced by the environmental conditions (6), growth rates are typically reported individually for the basal and prism faces (28, 29). Figure 3 gives an overview of the ice crystal axial dimensions measured during the seeding experiment at $-5.2^{\circ }$C for different growth times. Ice crystals with a major axis length of up to 500 μm and up to 800 μm were measured during S5-6.6 and S5-9.3, respectively. The major axis length of the ice crystals measured during S5-9.3 was on average 33% larger than the one observed during S5-6.6. A large variability was observed in the major axis length, while the minor axis varied less for both missions (Fig. 3b,c). The variability among the higher values of the ice crystal size distribution might even be underestimated due to sedimentation of larger ice crystals (Fig. 2a). The variability in the major axis length at $-5.2^{\circ }$C is likely linked to varying levels of supersaturation experienced by the ice crystals during their growth, as discussed in the following paragraphs. The ice crystals measured at $-5.2^{\circ }$C were characterized by a mean aspect ratio of 3.1±1.3 (Fig. 3a) and a columnar shape (Fig. S3A). On the other hand, the ice crystals observed during the seeding missions at $-7.2^{\circ }$C exhibited an aspect ratio of 1.6±0.3 (Fig. S2A). The ice crystal images showed the occurrence of plates and short columns including a large number of hollow columns indicative of high supersaturation (Fig. S3B).

**Fig. 4.**
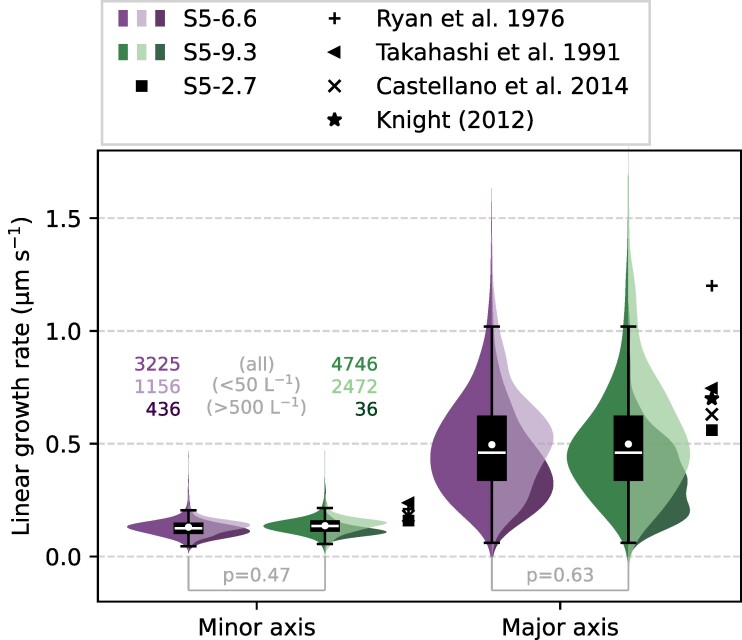
Linear growth rates of ice crystals along the minor and major axes are shown for seeding missions S5-6.6 (purple) and S5-9.3 (green). The left halves of the violin plots illustrate the mean distribution including all data, while the right halves of the violin plots show the mean distribution within stable mixed-phase regions (≤ 50 L^−1^, light color) and rapid glaciating regions (≥ 500 L^−1^, dark color), with the ice crystal number concentration averaged over a 1 s interval. The colored numbers represent the number of time steps included in the respective half violin plots. Black markers denote the linear growth rates reported in the specified studies (Ryan et al. (28): $T=-5.0^{\circ }$C, t=2.5 min; Takahashi et al. (29): $T=-5.3^{\circ }$C, t=5 min; Castellano et al. (39): $\text{T}=-6.5^{\circ }$C, t=4.5 min; Knight (40): $\text{T}=-5.0^{\circ }$C, t=40 min). Additionally, the linear growth rate computed from the difference between the seeding missions (S5-2.7) is shown as a black square (see Methods section). A Kolmogorov–Smirnov test was conducted at a significance level of 0.05 to evaluate whether the distribution of growth rates measured during S5-6.6 and S5-9.3 were significantly different; a *P*-value ≤ 0.05 indicates a significant difference. The test was performed on 500 independent subsamples, each with a sample size of 100. The mean *P*-values across these subsamples are reported, with rejection rates of 7% for the minor axis and 3% for the major axis.

The diffusional growth of ice crystals can be expressed as the growth of the axial dimensions as a function of time. While Ryan et al. (41) found that the ice crystal growth can be adequately described as a linear function of time (i.e. constant growth rate) at short time scales (<3 min), Takahashi et al. (29) found a power-law function more suitable for longer time periods (3–30 min). In the following, we evaluate whether the growth of the observed ice crystal populations can be represented by a constant growth rate, i.e. independent of time. At $-5.2^{\circ }$C, an average linear growth rate of 0.13 ± 0.038 μm s^−1^ was observed for the minor axis and 0.50 ± 0.23 μm s^−1^ for the major axis (Fig. 4), whereas the average linear growth rates at $-7.2^{\circ }$C were 0.17 ± 0.050 μm s^−1^ for the minor axis and 0.28 ± 0.087 μm s^−1^ for the major axis (Fig. S4). While columnar growth dominated the seeding missions at $-5.2^{\circ }$C, a simultaneous presence of columnar and plate-like growth patterns (resulting in either short columns or plates) was observed for the missions at $-7.2^{\circ }$C, in line with previous studies (6, 29, 41). Interestingly, no significant difference was observed for the average growth rates measured during S5-6.6 and S5-9.3 (Fig. 4), as indicated by a Kolmogorov–Smirnov test (P≥ 0.05). Moreover, the growth rates computed from the difference between consecutive seeding missions (i.e. S5-2.7, see Methods section) was in good agreement with the respective seeding missions. These findings suggest that at $-5.2^{\circ }$C, the observed ice crystal populations grew approximately linearly over time (i.e. constant growth rate) during the time period of 6.6 to 9.2 min. This also aligns with the laboratory study of Knight (40), who recorded long time series, lasting up to several hours, of individually grown ice crystals at $-5^{\circ }$C and found nearly constant growth rates over time for needles.

The average linear growth rates observed in Figs. 4 and S4 were notably lower compared to the linear growth rates documented in earlier laboratory studies (28, 29, 39) and showed a substantial variability. Nevertheless, the largest measured ice crystals exhibited comparable or even higher growth rates than those reported in previous studies. Considering the high ice crystal number concentrations measured within the seeding plume (Figs. 2c and S1C), the variability in the observed linear growth rates and the lower averages compared to earlier studies can partly be attributed to the competition among the ice crystals for the limited available water vapor. Indeed, a depletion of the liquid phase was observed during the seeding missions, particularly in time periods with high ice crystal number concentrations, with certain cloud regions experiencing complete glaciation (Figs. 2c,d and S1C,D). To account for that, we investigated the growth rates of ice crystal populations for different mixed-phase conditions, specifically exploring the growth rates within stable mixed-phase regions and rapid glaciating regions. Based on the mean ice crystal number concentrations and glaciation times reported in Korolev et al. (42), our data were divided into two regimes using an ice crystal concentration threshold of below 50 L^−1^ to define “stable mixed-phase regions” (i.e. vapor unlimited regime) and above 500 L^−1^ to define “rapid glaciating regions” (i.e. vapor-limited regime). The separation into these regions shows that at $-5.2^{\circ }$C, the growth rates along the major axis are significantly lower in the rapid glaciating regions (0.38 ± 0.18 μm s^−1^) compared to the stable mixed-phase regions (0.53 ± 0.24 μm s^−1^), as indicated by the Kolmogorov–Smirnov test (P≤0.05). This suggests that the number concentration of ice crystals and thus the competition for the limited available water vapor can significantly reduce the supersaturation and ice crystal growth rates. Indeed, direct numerical simulations conducted by Chen et al. (43) confirm that fluctuations in the small-scale supersaturation field in the immediate surrounding of ice crystals broaden the ice crystal size distribution, particularly in environments close to ice saturation.

**Fig. 5.**
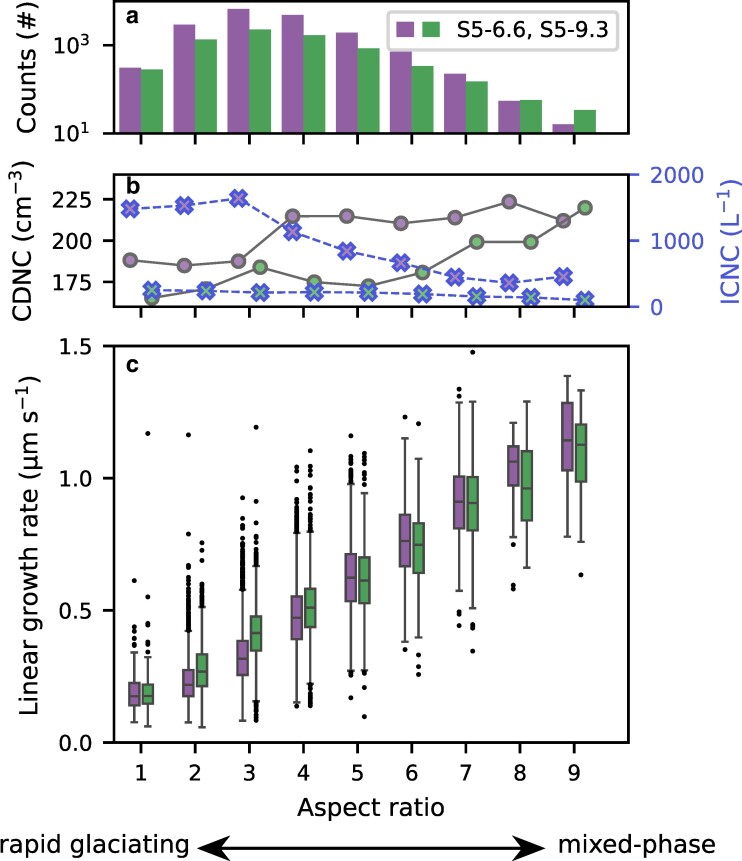
Linear growth rates of ice crystals along the major axis are shown for seeding missions S5-6.6 (purple) and S5-9.3 (green) as a function of aspect ratio. a) The histograms show the number of counts in the respective aspect ratio bin. b) Mean cloud droplet number concentration (CDNC, solid gray lines) and mean ice crystal number concentration (ICNC, dashed blue lines) measured within each aspect ratio bin. c) The boxplots show the linear growth rate along the major axis. As depicted by the black arrow, the aspect ratio is used as a proxy for supersaturation experienced during ice growth, where a low aspect ratio corresponds to low supersaturation (rapid glaciating region), while a high aspect ratio corresponds to high supersaturation (stable mixed-phase region). It is important to note that other factors (e.g. temperature fluctuations) can also influence the aspect ratio.

To investigate the impact of the supersaturation on the growth rates of individual ice crystals, we used the aspect ratio at $-5.2^{\circ }$C as a proxy for the supersaturation, where a high aspect ratio is assumed to be an indicator for high supersaturation experienced by the ice crystals during growth. While fluctuations in the supersaturation do not change the basic habit of ice crystals (e.g. column vs. plate), it influences how fast the preferred axis grows (15). Indeed, as depicted in Fig. 5b, ice crystals with a larger aspect ratio were observed at $-5.2^{\circ }$C when lower ice crystal number concentrations and higher cloud droplet number concentrations were present. These ice crystals likely experienced higher supersaturation during growth, consistent with previous laboratory studies (44, 45) that observed faster ice growth in the presence of higher liquid water content. This accelerated growth was attributed to the repeated close proximity of supercooled cloud droplets, which enhanced rapid vapor transport from the cloud droplets to the ice crystals ((44), “vapor flush” effect). As a result, the observed growth rates (1.03 ± 0.16 μm s^−1^, Fig. 5c) align more closely or even exceed those reported in previous laboratory studies and can be considered representative of conditions in which ice crystal growth is not vapor-limited or in which cloud droplets and ice crystals are uniformly distributed within a cloud volume ((23), genuinely mixed). On the other hand, when considering ice crystals with lower aspect ratio, the ice crystal growth rates were significantly lower (0.24 ± 0.090 μm s^−1^) and likely limited by water vapor. This case can be important in regions with high ice crystal number concentrations, such as those with strong secondary ice production (46, 47) or in mixed-phase regions in which cloud droplets and ice crystals are spatially separated ((23), conditionally mixed).

The observed variability in ice crystal growth rates in natural clouds, and consequently in ice crystal sizes and fall speeds, can potentially accelerate the onset of precipitation initiation through more efficient aggregation (48) and secondary ice production from ice–ice collisions (49). Quantifying the variability in ice crystal growth rates and identifying the conditions that influence them has significant implications for predicting the amount and spatial distribution of precipitation, particularly in shallow clouds and in regions with complex terrain (50, 51).

## Conclusion

In this study, we repurposed weather modification for fundamental cloud research by conducting repeated glaciogenic cloud seeding experiments in persistent supercooled stratus clouds to infer ice crystal growth rates in natural clouds. Seeding material was released from a multirotor UAV and the seeding-induced microphysical changes were measured 4–10 min downwind by ground-based remote sensing and in situ instrumentation on a tethered balloon system. Seeding signatures were detected by increased radar reflectivities, along with elevated ice crystal number concentrations and a simultaneous reduction in the cloud droplet number concentration up to complete glaciation in single cloud patches. This emphasizes the efficiency of the WBF process in producing precipitation-sized particles (≥100μm) within a few minutes, highlighting its importance for precipitation initiation.

The seeding distance and consequently the ice growth time were systematically adjusted between consecutive seeding missions, while the environmental conditions remained approximately constant, enabling comprehensive ice growth studies within a quasi-steady-state natural environment. At $-5.2^{\circ }$C, the seeding experiments revealed columnar growth with an average growth rate of 0.50 ± 0.23 μm s^−1^ along the major axis, while a transition to short columns and plates was observed at $-7.2^{\circ }$C, characterized by an average growth rate of 0.28 ± 0.087 μm s^−1^. Our findings indicate that the ice crystal populations in natural clouds grew nearly linearly over time at $-5.2^{\circ }$C within the observed time scales (6–10 min). Further investigations over a broader range of time and ambient conditions are required to thoroughly characterize the temporal (in)dependence of growth rates. In-depth knowledge of ice growth processes and the ways they are represented in numerical weather prediction models directly impacts the precipitation forecast skill (e.g. extreme precipitation).

Our observations show large variability in ice crystal growth rates within natural clouds, underscoring their sensitivity to both ice crystal number concentrations and supersaturation. Specifically, in rapid glaciating regions characterized by high ice crystal number concentrations, the linear growth rates at $-5.2^{\circ }$C (0.38 ± 0.18 μm s^−1^) were significantly lower compared to those in water vapor unlimited regions (0.53 ± 0.24 μm s^−1^), as the ice crystals in rapid glaciating regions likely experienced lower supersaturation in their immediate vicinity during growth. This variability in ice crystal growth rates potentially accelerates precipitation initiation, with significant implications for both precipitation forecast and climate simulations. Thus, we recommend that future research focuses on investigating ice crystal growth rates under conditions of fluctuating supersaturation and/or temperature, using well-designed field experiments or innovative laboratory approaches.

This study provides unambiguous evidence that the targeted use of weather modification techniques can be employed to study microphysical processes in natural clouds, specifically enabling the investigation of ice crystal growth rates at temperatures above $-10^{\circ }$C—a pursuit previously limited in field studies due to the scarcity of naturally occurring ice nucleating particles at these high temperatures. Future work aims to extend this analysis to different environmental and microphysical conditions, leveraging these observations to constrain and validate model simulations. In particular, these comprehensive observations can help to develop more realistic parameterizations for weather and climate models, extending beyond what is achievable in a laboratory setting. Overall, this study shows the versatile use of weather modification, not only for precipitation enhancement and climate intervention but also for advancing our understanding of cloud processes (e.g. ice growth processes, aerosol-cloud interactions), validating remote sensing retrievals, and refining model parameterizations.

## Materials and methods

### CLOUDLAB seeding experiments

In the framework of the CLOUDLAB project (35), targeted glaciogenic cloud seeding experiments were conducted in supercooled stratus clouds. These experiments were performed in a restricted airspace in the Swiss Plateau near Eriswil, Switzerland (47°04′14″N, 7°52′22″E), which is embedded between the Alps and Jura mountains and often covered by persistent wintertime stratus clouds (37). The principle behind glaciogenic cloud seeding is based on the concept that natural precipitation is limited by the number of ice crystals and thus ice nucleating particles, and that the injection of additional ice nucleating particles into supercooled clouds can trigger ice and subsequent precipitation formation (52). In contrast to earlier seeding initiatives primarily aimed at enhancing precipitation (38, 52–54), CLOUDLAB employs seeding as a method to further our understanding of the fundamental processes governing ice formation and growth. The seeding material was injected into supercooled stratus clouds from a burn-in-place flare mounted on a UAV which flew horizontal tracks perpendicular to the wind 1–4 km upwind of the observational site (55). The seeding-induced microphysical changes were observed by an extensive set of ground-based remote sensing and in situ instrumentation downstream of the seeding location. By repeating seeding experiments in this quasi-steady-state environment, with systematic adjustment of one experimental parameter (i.e. ice crystal growth time), we were able to perform laboratory-like studies on ice crystal growth in natural clouds.

### Instrumentation

The seeding material was introduced into the cloud by a multirotor UAV (Meteodrone MM-670, Meteomatics AG), which has a propeller heating mechanism to enable flight in supercooled clouds with icing conditions. The UAV was equipped with one burn-in-place flare (55, 56), which contains a mixture of silver iodide, silver chloride, ammonium salt, and potassium salt (Zeus MK2, Cloud Seeding Technologies). One seeding flare has a burning time of around 5 min. The flight pattern of the UAV (i.e. autonomous mission) was determined before each seeding mission based on the wind measurements from a radar wind profiler (LAP-3000, Vaisala) installed at the measurement site.

A 94 GHz (W-band) frequency-modulated continuous wave Doppler cloud radar (FMCW-94-DP, RPG, (57)) and a 35 GHz (Ka-band) pulsed, polarimetric Doppler radar (Mira-36, Metek, (58)) were deployed at the observational site to measure the background cloud and seeding signatures. The FMCW-94-DP was operated in vertical pointing mode, whereas the Mira-36 performed Range Height Indicator (RHI) scans perpendicular to the main wind direction or Plan Position Indicator (PPI) scans at a fixed elevation angle downwind of the measurement site. The scans were conducted at a speed of 1°/s.

The tethered balloon system HoloBalloon equipped with the holographic imager HOLIMO (36) provided co-located in situ measurements of the phase-resolved cloud properties, which complemented the radar observations. HOLIMO images an ensemble of cloud particles in the size range between 6 μm and 2 mm in a 3D sample volume (36, 59). Here, we used a sample volume of 26.95 cm^3^ for the ice crystal growth analysis and a smaller sample volume of 11.76 cm^3^ for observing the cloud microphysical properties (e.g. Figs. 2 and S1) due to instrumental limitations (e.g. sizing small particles). The HOLIMO data were analyzed with a frame rate of 20 Hz during seeding conditions and of 10 Hz during background conditions.

The images captured by HOLIMO were automatically classified into cloud droplets (circular) and ice crystals (noncircular) based on the particle shape using a neural network (60). As in Ramelli et al. (61), differentiation between cloud droplets and ice crystals was done for particles larger than 25 μm in major axis diameter due to resolution limitations (i.e. all particles smaller than 25 μm in diameter were classified as cloud droplets). After the automated classification, the ice crystals were manually classified into pristine and aggregated ice crystals based on the particle shape. This results in the following uncertainties: cloud droplet number concentrations (5%), ice crystal number concentration for ice crystals smaller than 100 μm (15%), ice crystal number concentration for ice crystals larger than 100 μm (5–10%) (61, 62). The liquid and ice water contents were derived from the size distributions measured by HOLIMO, with the ice water content calculated using the mass-size relationship from Cotton et al. (63).

### Inferring ice crystal growth rates from seeding experiments

In this study, a linear ice crystal growth rate was derived from the seeding experiments by dividing the observed size dimension by the corresponding growth time. For that, it was essential to precisely characterize both the size and growth time of the measured ice crystals. To size the ice crystals, we analyzed the minor and major axis dimensions of the pristine ice crystals detected by HOLIMO. Aggregated ice crystals were not considered in the growth analysis. The uncertainty in sizing ice crystals was estimated at 20% for ice crystals with a diameter of 25 μm and 5–10% for ice crystals larger than 100 μm (62). An additional uncertainty of 10% is introduced due to uncertainty of the orientation of the ice crystals with respect to the camera plane, resulting in a combined size uncertainty of 15% for ice crystals larger than 100 μm.

The ice crystal growth time, spanning from nucleation to detection, was estimated by considering observations from multiple data sources; e.g. (i) dividing the seeding distance by the wind speed measured by the wind profiler or (ii) considering the time between the seeding flare ignition and (a) the first appearance of ice crystals in the vertically pointing cloud radar, (b) the first appearance of ice crystals in HOLIMO, and (c) the first appearance of seeding particles in the Portable Optical Particle Spectrometer (POPS, Handix Scientific) mounted on HoloBalloon. Following these approaches, the growth time could be estimated with an uncertainty of 10%. The estimation of the growth time was based on the assumption that most ice crystals nucleated immediately after the release of the seeding material. This is supported by the high ice nucleating activity of the seeding particles at temperatures below −5 °C previously observed in the laboratory (64). After the initial nucleation event, the high concentration of ice crystals reduces the ambient saturation ratio, which might then become too low to support further nucleation. The uncertainty associated with the nucleation time can be reduced by comparing observations from consecutive seeding missions where the ice crystals underwent the same nucleation process (as done in Figs. 4 and S4). This was achieved by computing an average linear growth rate based on two successive seeding missions by taking the difference in median ice crystal dimensions (e.g. S5-6.6 and S5-9.3) and dividing it by the corresponding difference in growth time (e.g. S5-2.7). The good agreement between the computed linear growth rates from the intermission comparison with the linear growth rates observed in individual missions (Figs. 4 and Fig. S4) further supports the assumption that most ice crystals nucleated immediately after seeding. However, we cannot rule out the possibility of further nucleation events downstream of the seeding location. Continued nucleation could explain some of the variability observed in the lower range of growth rates. Overall, considering the uncertainties in both size and growth time, the linear growth rate was inferred with a 20% uncertainty.

## Supplementary Material

pgae402_Supplementary_Data

## Data Availability

Data are available at https://doi.org/10.5281/zenodo.13712737 (65). Analysis and plotting scripts are available at https://doi.org/10.5281/zenodo.13715279 (66).

## References

[1] Mülmenstädt J , Sourdeval O, Delanoë J, Quaas J. 2015. Frequency of occurrence of rain from liquid-, mixed-, and ice-phase clouds derived from a-train satellite retrievals. Geophys Res Lett. 42:6502–6509.

[2] Heymsfield AJ , *et al*. 2020. Contributions of the liquid and ice phases to global surface precipitation: observations and global climate modeling. J Atmo Sci. 77:2629–2648.

[3] Matus AV , L’Ecuyer TS. 2017. The role of cloud phase in earth’s radiation budget. J Geophys Res Atmos. 122:2559–2578.

[4] Korolev A , *et al*. 2017. Mixed-phase clouds: progress and challenges. Meteorol Monogr. 58:5.1–5.50.

[5] Mitchell DL , Zhang R, Pitter RL. 1990. Mass-dimensional relationships for ice particles and the influence of riming on snowfall rates. J Appl Meteorol Climatol. 29:153–163.

[6] Bailey MP , Hallett J. 2009. A comprehensive habit diagram for atmospheric ice crystals: confirmation from the laboratory, AIRS II, and other field studies. J Atmos Sci. 66:2888–2899.

[7] Morrison H , *et al*. 2020. Confronting the challenge of modeling cloud and precipitation microphysics. J Adv Modeling Earth Syst. 12:e2019MS001689. 10.1029/2019MS001689 PMC7507216 32999700

[8] Kanji ZA , *et al*. 2017. Overview of ice nucleating particles. Meteorol Monogr. 58:1.1–1.33.

[9] Frank FC . 1982. Snow crystals. Contemp Phys. 23:3–22.

[10] Hueholt DM , Yuter SE, Miller MA. 2022. Revisiting diagrams of ice growth environments. Bull Am Meteorol Soc. 103:E2584–E2603.

[11] Sazaki G , Zepeda S, Nakatsubo S, Yokomine M, Furukawa Y. 2012. Quasi-liquid layers on ice crystal surfaces are made up of two different phases. Proc Natl Acad Sci U S A. 109:1052–1055. 22232653 10.1073/pnas.1116685109 PMC3268306

[12] Mason BJ , Bryant GW, den Heuvel APV. 1963. The growth habits and surface structure of ice crystals. Phil Magaz J Theoret Exp Appl Phys. 8:505–526.

[13] Nelson J , Knight C. 1998. Snow crystal habit changes explained by layer nucleation. J Atmos Sci. 55:1452–1465.

[14] Hallett J , Mason BJ, Bernal JD. 1958. The influence of temperature and supersaturation on the habit of ice crystals grown from the vapour. Proc R Soc Lond Ser A. 247:440–453.

[15] Kobayashi T . 1961. The growth of snow crystals at low supersaturations. Phil Magaz J Theoret Exp Appl Phys. 6:1363–1370.

[16] Libbrecht KG . 2017. Physical dynamics of ice crystal growth. Annu Rev Mater Res. 47:271–295.

[17] Harrington JY , Pokrifka GF. 2024. An approximate criterion for morphological transformations in small vapor grown ice crystals. J Atmos Sci. 81:401–416.

[18] Korolev A . 2007. Limitations of the Wegener–Bergeron–Findeisen mechanism in the evolution of mixed-phase clouds. J Atmos Sci. 64:3372–3375.

[19] Storelvmo T , Tan I. 2015. The Wegener-Bergeron-Findeisen process - its discovery and vital importance for weather and climate. Meteorol Zeitschrift. 24:455–461.

[20] Wegener A . 1911. Thermodynamik der atmosphäre. JA Barth.

[21] Bergeron T . 1935. On the physics of clouds and precipitation. Proc 5th Assembly UGGI Lisbon. 2:156–180.

[22] Findeisen Z . 1938. Kolloid meteorologische vorgange bei neiderschlags-bildung. Meteorol Zeitschrift. 55:121.

[23] Korolev A , Milbrandt J. 2022. How are mixed-phase clouds mixed? Geophys Res Lett 49:e2022GL099578. 10.1029/2022GL099578 PMC9540255 36246738

[24] Korolev A , Leisner T. 2020. Review of experimental studies of secondary ice production. Atmos Chem Phys. 20:11767–11797.

[25] Hofer S , *et al*. 2023. Realistic representation of mixed-phase clouds increases future climate warming. PREPRINT (Version 1) available at Research Square.

[26] Omanovic N , *et al*. 2024. Evaluating the Wegener–Bergeron–Findeisen process in icon in large-eddy mode with in situ observations from the cloudlab project. Atmos Chem Phys. 24:6825–6844.

[27] Koenig LR . 1971. Numerical modeling of ice deposition. J Atmos Sci. 28:226–237.

[28] Ryan BF , Wishart ER, Shaw DE. 1976. The growth rates and densities of ice crystals between -3°C and -21°C. J Atmos Sci. 33:842–850.

[29] Takahashi T , Endoh T, Wakahama G, Fukuta N. 1991. Vapor diffusional growth of free-falling snow crystals between -3 and -23 C. J Meteorol Soc Jpn Ser II. 69:15–30.

[30] Field PR . 1999. Aircraft observations of ice crystal evolution in an altostratus cloud. J Atmos Sci. 56:1925–1941.

[31] Kenneth Lo K , Passarelli RE. 1982. The growth of snow in winter storms: an airborne observational study. J Atmos Sci. 39:697–706.

[32] Mitchell DL . 1988. Evolution of snow-size spectra in cyclonic storms. Part I: Snow growth by vapor deposition and aggregation. J Atmos Sci. 45:3431–3451.

[33] Heymsfield AJ , *et al*. 2011. Ice in clouds experiment-layer clouds. Part I: Ice growth rates derived from lenticular wave cloud penetrations. J Atmos Sci. 68:2628–2654.

[34] Baker BA , Lawson RP. 2006. In situ observations of the microphysical properties of wave, cirrus, and anvil clouds. Part I: Wave clouds. J Atmos Sci. 63:3160–3185.

[35] Henneberger J , *et al*. 2023. Seeding of supercooled low stratus clouds with a UAV to study microphysical ice processes: an introduction to the CLOUDLAB project. Bull Am Meteorol Soc. 104:E1962–E1979.

[36] Ramelli F , Beck A, Henneberger J, Lohmann U. 2020. Using a holographic imager on a tethered balloon system for microphysical observations of boundary layer clouds. Atmos Meas Tech. 13:925–939.

[37] Scherrer SC , Appenzeller C. 2014. Fog and low stratus over the Swiss plateau - a climatological study. Int J Climatol. 34:678–686.

[38] French JR , *et al*. 2018. Precipitation formation from orographic cloud seeding. Proc Natl Acad Sci U S A. 115:1168–1173. 29358387 10.1073/pnas.1716995115 PMC5819430

[39] Castellano NE , Ávila EE, Bürgesser RE, Saunders CP. 2014. The growth of ice particles in a mixed phase environment based on laboratory observations. Atmos Res. 150:12–20.

[40] Knight CA . 2012. Ice growth from the vapor at 5°C. J Atmos Sci. 69:2031–2040.

[41] Ryan BF , Wishart ER, Holroyd EW. 1974. The densities and growth rates of ice crystals between -5C and -9C. J Atmos Sci. 31:2136–2141.

[42] Korolev A , Isaac G. 2003. Phase transformation of mixed-phase clouds. Q J R Meteorol Soc. 129:19–38.

[43] Chen S , *et al*. 2023. Mixed-phase direct numerical simulation: ice growth in cloud-top generating cells. Atmos Chem Phys. 23:5217–5231.

[44] Song N , Lamb D. 1994. Experimental investigations of ice in supercooled clouds. Part 1: System description and growth of ice by vapor deposition. J Atmos Sci. 51:91–103.

[45] Takahashi T . 2014. Influence of liquid water content and temperature on the form and growth of branched planar snow crystals in a cloud. J Atmos Sci. 71:4127–4142.

[46] Korolev A , *et al*. 2020. A new look at the environmental conditions favorable to secondary ice production. Atmos Chem Phys. 20:1391–1429.

[47] Pasquier JT , *et al*. 2022. Conditions favorable for secondary ice production in arctic mixed-phase clouds. Atmos Chem Phys. 22:15579–15601.

[48] Pruppacher HR , Klett JD, Wang PK. 1998. Microphysics of clouds and precipitation. Taylor & Francis.

[49] Vardiman L . 1978. The generation of secondary ice particles in clouds by crystal–crystal collision. J Atmos Sci. 35:2168–2180.

[50] Colle BA , Zeng Y. 2004. Bulk microphysical sensitivities within the mm5 for orographic precipitation. Part I: The Sierra 1986 event. Mon Weather Rev. 132:2780–2801.

[51] Woods CP , Stoelinga MT, Locatelli JD. 2007. The improve-1 storm of 1–2 February 2001. Part III: Sensitivity of a mesoscale model simulation to the representation of snow particle types and testing of a bulk microphysical scheme with snow habit prediction. J Atmos Sci. 64:3927–3948.

[52] Bruintjes RT . 1999. A review of cloud seeding experiments to enhance precipitation and some new prospects. Bull Am Meteorol Soc. 80:805–820.

[53] Flossmann AI , *et al*. 2019. Review of advances in precipitation enhancement research. Bull Am Meteorol Soc. 100:1465–1480.

[54] Dessens J , Sánchez J, Berthet C, Hermida L, Merino A. 2016. Hail prevention by ground-based silver iodide generators: results of historical and modern field projects. Atmos Res. 170:98–111.

[55] Miller AJ , et al 2024. Two new multirotor UAVs for glaciogenic cloud seeding and aerosol measurements within the cloudlab project. Atmos Meas Tech. 2024:1–25.

[56] Miller AJ , *et al*. 2024. Multirotor UAV icing correlated to liquid water content measurements in natural supercooled clouds. Cold Reg Sci Technol. 225:104262.

[57] Küchler N , *et al*. 2017. A w-band radar–radiometer system for accurate and continuous monitoring of clouds and precipitation. J Atmos Ocean Tech. 34:2375–2392.

[58] Görsdorf U , *et al*. 2015. A 35-GHz polarimetric Doppler radar for long-term observations of cloud parameters-description of system and data processing. J Atmos Ocean Tech. 32:675–690.

[59] Henneberger J , Fugal JP, Stetzer O, Lohmann U. 2013. Holimo II: a digital holographic instrument for ground-based in situ observations of microphysical properties of mixed-phase clouds. Atmos Meas Tech. 6:2975–2987.

[60] Touloupas G , Lauber A, Henneberger J, Beck A, Lucchi A. 2020. A convolutional neural network for classifying cloud particles recorded by imaging probes. Atmos Meas Tech. 13:2219–2239.

[61] Ramelli F , *et al*. 2021. Microphysical investigation of the seeder and feeder region of an alpine mixed-phase cloud. Atmos Chem Phys. 21:6681–6706.

[62] Beck A . 2017. Observing the microstructure of orographic clouds with HoloGondel [PhD thesis]. ETH Zurich.

[63] Cotton RJ , *et al*. 2013. The effective density of small ice particles obtained from in situ aircraft observations of mid-latitude cirrus. Q J R Meteorol Soc. 139:1923–1934.

[64] Chen J , Rösch C, Rösch M, Shilin A, Kanji ZA. 2024. Critical size of silver iodide containing glaciogenic cloud seeding particles. Geophys Res Lett. 51:e2023GL106680.

[65] Ramelli F , et al 2024. Data for the publication “Repurposing weather modification for cloud research showcased by ice crystal growth”. 10.5281/zenodo.13712737. PMC11423147 39323981

[66] Ramelli F , *et al*. 2024. Software for the publication “Repurposing weather modification for cloud research showcased by ice crystal growth”. 10.5281/zenodo.13715279. PMC11423147 39323981

