# Supplementary material for: Repurposing weather modification for cloud research showcased by ice crystal growth
Source: PNAS Nexus. 2024 Sep 18;3(9):pgae402. doi: 10.1093/pnasnexus/pgae402 (PMC11423147; doi:10.1093/pnasnexus/pgae402)
Supplement: pgae402_Supplementary_Data [file pgae402_supplementary_data.pdf]

Supporting Information for

Repurposing weather modification for cloud research showcased by ice crystal growth

Fabiola Ramelli, Jan Henneberger, Christopher Fuchs, Anna J. Miller, Nadja Omanovic, Robert Spirig, Huiying Zhang, Robert O. David, Kevin Ohneiser, Patric Seifert, and Ulrike Lohmann

Fabiola Ramelli and Jan Henneberger.
E-mail: fabiola.ramelli@env.ethz.ch, jan.henneberger@env.ethz.ch

This PDF file includes:

Figs. S1 to S4
SI References

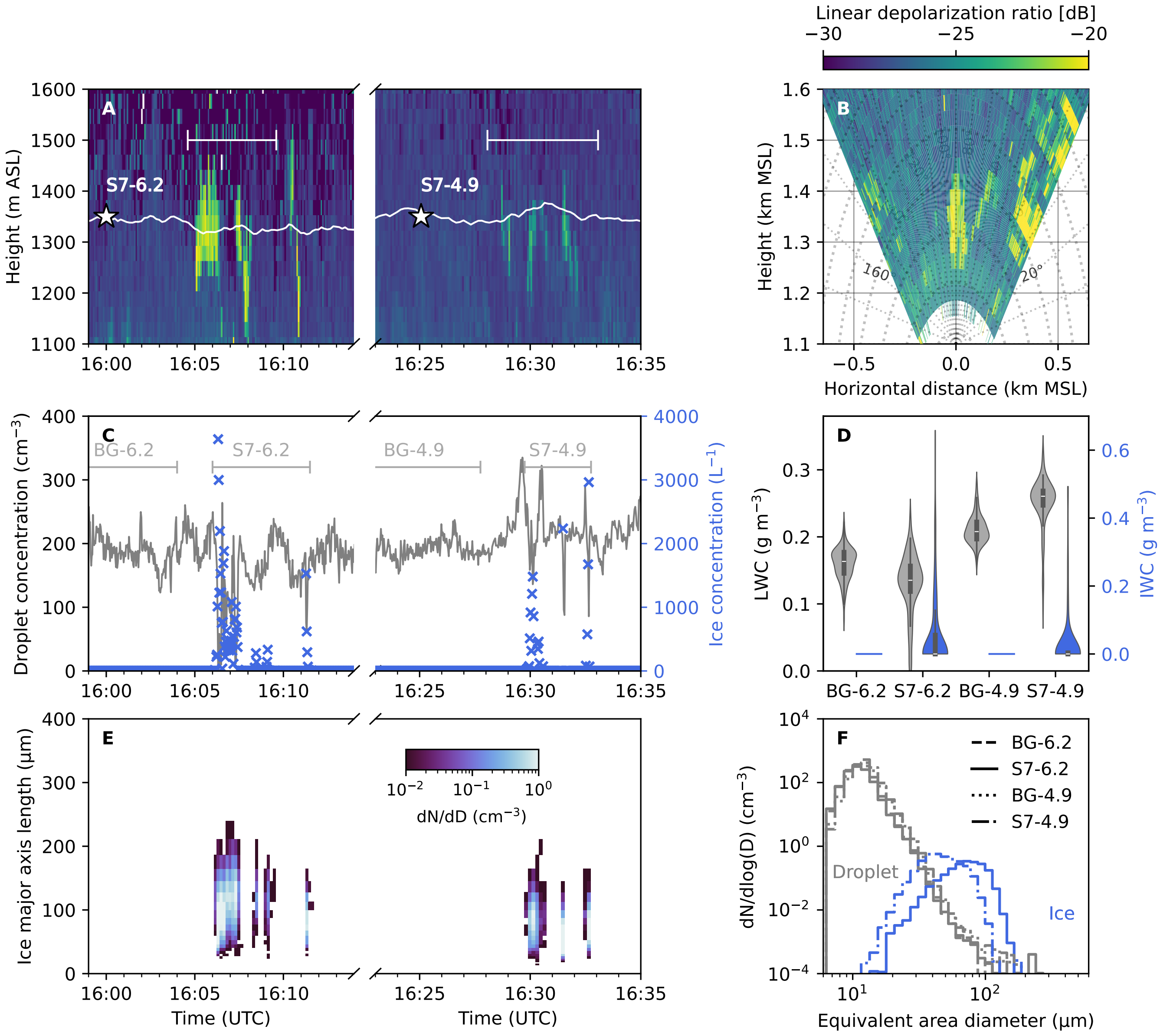

Fig. S1. Temporal evolution of the microphysical properties observed during the seeding missions S7-6.2 and S7-4.9 conducted on 27 January 2023 at a seeding temperature of -7.2 °C and at seeding distances of 2 km and 3 km, respectively. (A) Time–height linear depolarization ratio measured by the vertically-pointing W-band cloud radar. The white line shows the height of the tethered balloon, whereas the white stars indicate the height and start time of the seeding missions. The black bars highlight the time periods of expected seeding signal, taking into account the mean wind speed and the flare burning time of 5 min. (B) Linear depolarization ratio measured by the scanning Ka-band cloud radar during a range height indicator (RHI) scan at 16:05 UTC. (C) Timeseries of the cloud droplet number concentration (grey) and ice crystal number concentration (blue). The background periods (BG-6.2: 15:54:00 - 16:04:00 UTC, BG-4.9: 16:17:45 - 16:27:45 UTC) and seeding periods (S7-6.2: 16:06:00 - 16:11:30 UTC, S7-4.9: 16:29:45 - 16:32:45 UTC) are highlighted by the horizontal grey bar. (D) Violin plots of the liquid water content (LWC, grey) and ice water content (IWC, blue) measured during the background and seeding periods (same periods as depicted in C). Note that no ice crystals were measured during the background periods (i.e. IWC = 0). (E) Timeseries of the ice crystal size distributions in terms of the major axis length. (F) Cloud droplet size distribution (grey) and ice crystal size distribution (blue) measured during BG-6.2 (dashed), S7-6.2 (solid), BG-4.9 (dotted) and S7-4.9 (dot-dashed) in terms of the equivalent area diameter. The data presented in C-F were obtained from HOLIMO and averaged over 2 s (C,D) and 10 s (E), respectively.

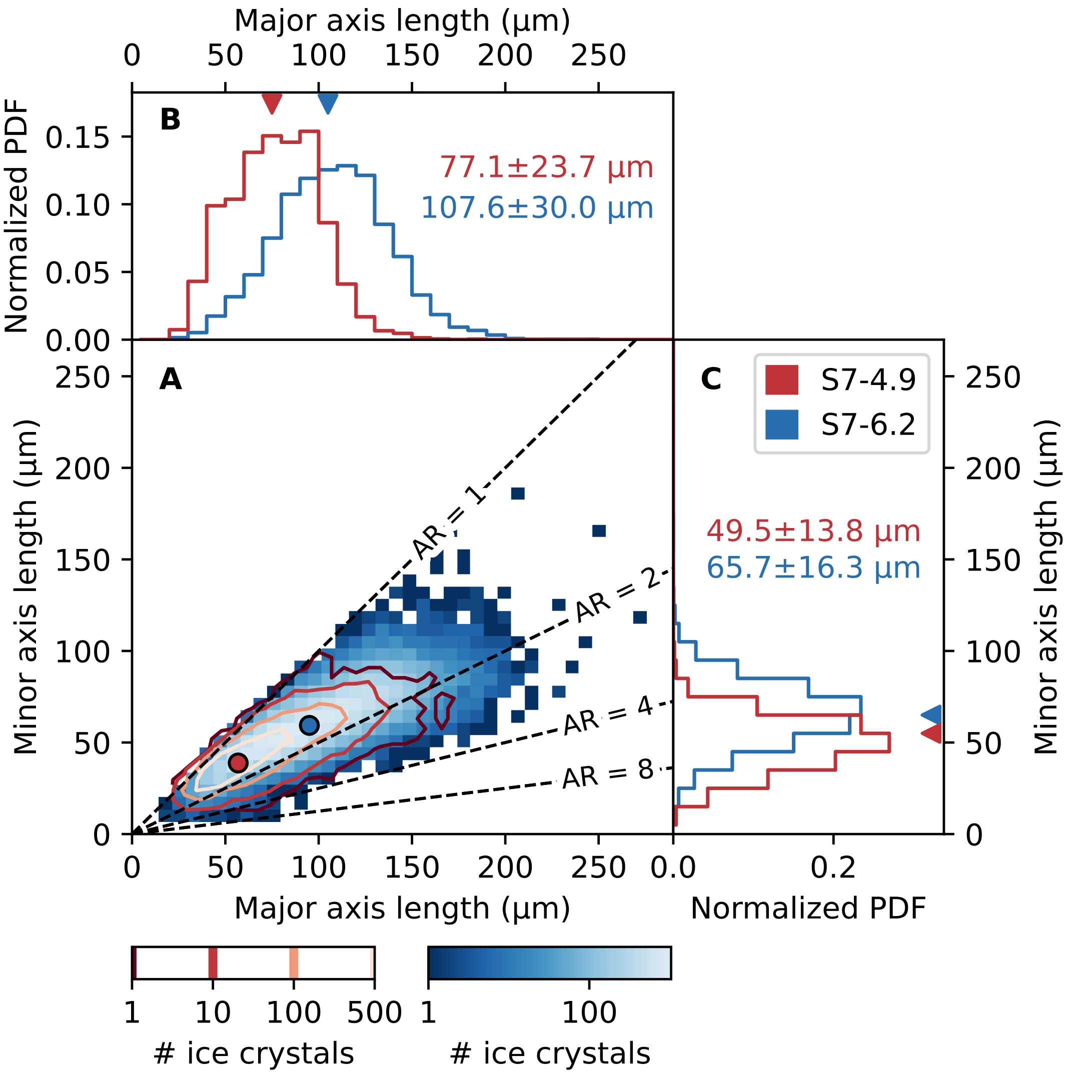

Fig. S2. Overview of the ice crystal axial dimensions measured during the seeding missions S7-4.9 (N=28945) and S7-6.2 (N=31222). (A) Two-dimensional histogram of the major and minor axis length of the detected ice crystals. The number of ice crystals observed with a given major and minor axis length during S7-6.2 are shown with the blue colormap, while S7-4.9 is shown by the red contour lines. The dots represent the corresponding median axial dimensions (including all observed ice crystals) and the black lines show contour lines of aspect ratio (defined by major axis length divided by minor axis length). (B, C) Normalized probability density function (PDF) of the major and minor axis length. The triangles represent the corresponding time-weighted medians, whereas the numbers indicate the mean values and standard deviations of the PDFs.

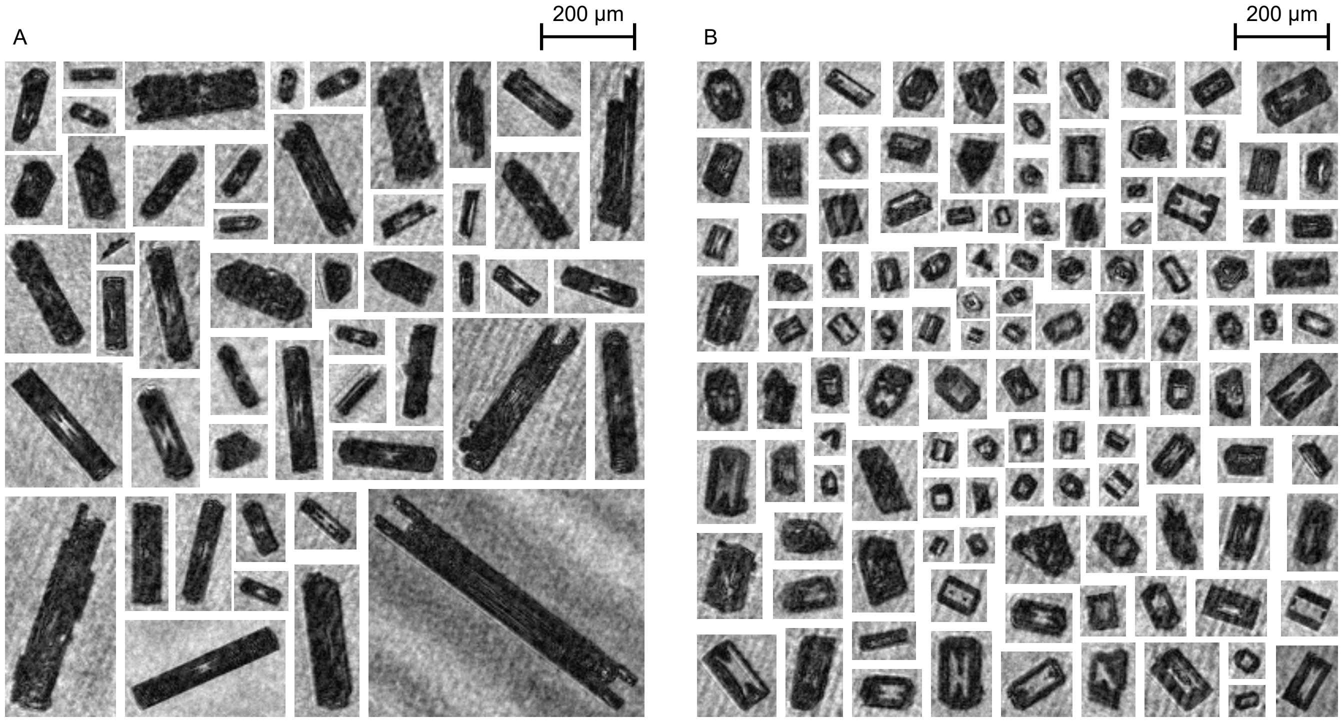

Fig. S3. A randomly selected sample of pristine ice crystal images observed by HOLIMO during (A) S5-6.4 and S5-9.1 and (B) S7-4.9 and S7-6.2.

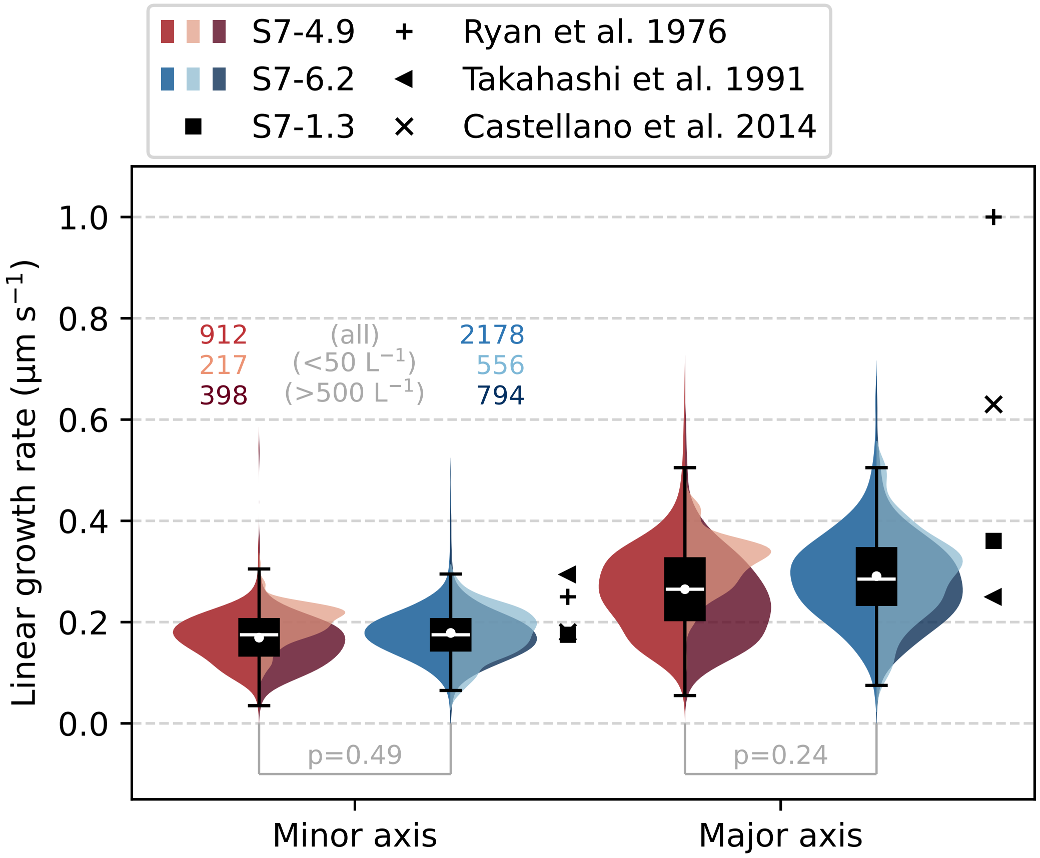

Fig. S4. Linear growth rates of ice crystals along the minor and major axes are shown for seeding missions S7-4.9 (red) and S7-6.2 (blue). The left halves of the violin plots illustrate the mean distribution including all data, while the right halves of the violin plots show the mean distribution within stable mixed-phase regions ($\leq$ 50 L^{-1}, light color) and rapid glaciating regions ($\geq$ 500 L^{-1}, dark color), with the ice crystal number concentration averaged over a 1 s interval. The colored numbers represent the number of time steps included in the respective half violin plots. Black markers denote the linear growth rates reported in the specified studies (Ryan et al. (1): T = -7.0 ℃, t = 2.5 min; Takahashi et al. (2): T = -8.6 ℃, t = 5 min; Castellano et al. (3): T = -6.5 ℃, t = 4.5 min). Additionally, the linear growth rate computed from the difference between the seeding missions (S7-1.3) is shown as a black square (see *Methods*). A Kolmogorov–Smirnov test was conducted at a significance level of 0.05 to evaluate whether the distribution of growth rates measured during S7-4.9 and S7-6.2 were significantly different; a p-value $\leq$ 0.05 indicates a significant difference. The test was performed on 500 independent subsamples, each with a sample size of 100. The mean p-values across these subsamples are reported, with rejection rates of 10% for the minor axis and 30% for the major axis.

References

1. BF Ryan, ER Wishart, DE Shaw, The growth rates and densities of ice crystals between -3°c and -21°c. *J. Atmospheric Sci.* **33**, 842 – 850 (1976).
2. T Takahashi, T Endoh, G Wakahama, N Fukuta, Vapor diffusional growth of free-falling snow crystals between -3 and -23 c. *J. Meteorol. Soc. Jpn. Ser. II* **69**, 15–30 (1991).
3. NE Castellano, EE Ávila, RE Bürgesser, CP Saunders, The growth of ice particles in a mixed phase environment based on laboratory observations. *Atmospheric Res.* **150**, 12–20 (2014).
